# Burden of gastrointestinal cancer in Asia; an overview 

**Published:** 2015

**Authors:** Mohamad Amin Pourhoseingholi, Mohsen Vahedi, Ahmad Reza Baghestani

**Affiliations:** 1 *Gastroenterology and Liver Diseases Research Center, Shahid Beheshti University of Medical Sciences, Tehran, Iran *; 2 *Department of Epidemiology and Biostatistics, School of Public Health, Tehran University of Medical Sciences, Tehran, Iran*; 3 *Department of Biostatistics, Shahid Beheshti University of Medical Sciences, Tehran, Iran *

**Keywords:** Gastrointestinal cancers, Burden, Asia

## Abstract

The cancers in the digestive system including gastric cancer, colorectal cancer, liver cancer, esophageal cancer and pancreatic cancer are one of the most common cancers in Asia. The burden of GI cancer is increasing in Asia because of aging, growth of the population and the risk factors including smoking, obesity, changing lifestyle and high prevalence of *H pylori,* HBV and HCV. In most Asian countries, cancer control programs or early detection and treatment services are limited despite this increase. There are many people in the developing countries inside Asia who have no health insurance and many of them are too poor to go for screening tests, early detection or medical treatments. Therefore, it is important for the health organizations and governments in each country to recognize these groups and reduce the incidence and mortality of gastrointestinal cancers, using simple and economic screening test, vaccination and changing risk factors such as smoking, diet and lifestyle by education programs.

## Introduction

Cancer is known as one of the major causes leading to many disorders, death, and disabilities worldwide (1). Among all organ cancers, gastrointestinal tract cancers (GI cancers) present an interesting pattern in distribution over the world. GI cancer is a term for the group of cancers that affect the digestive system, including gastric cancer (GC), colorectal cancer (CRC), hepatocellular carcinoma (HCC), esophageal cancer (EC) and pancreatic cancer (PC). Overall, the GI cancers are responsible for more cancers and more deaths from cancer than any other cancers. There is an increasing burden (incidence and mortality) in GI cancer worldwide and Asia is no exception (2).

Asia is the most populous continent in the world. Asia's population is raising faster than Europe or America and it covers approximately 4 billion people which hosts 60% of the world's current human population. According to information on the Lancet Asia Medical Forum website, the number of new cancer cases in Asia is set to increase from 3.5 million in 2002 to 8.1 million by 2020 if "current prevention and management strategies remain unchanged". The most common cancers in the digestive system including gastric cancer, colorectal cancer, liver cancer, esophageal cancer and pancreatic cancer are one of the most common cancers in Asia (3). 

In this brief review, we discussed the burden of these common GI cancers in Asia according to recently published studies on mortality, incidence and epidemiology of these cancers in Asian countries. Besides, the age-standardized rate (ASR) of incidence for Asia in 2012 (according to GLOBOCAN estimation) was employed in order to compare it with western countries**.**

**1. Gastric Cancer**

Gastric cancer (GC) is an important cause of mortality due to cancer (4) and is estimated to be one of the most leading causes of all deaths worldwide (5). 

Although the incidence of GC is decreasing, it is rarely detected early, and the prognosis remains poor. The majority of GC shows distant metastasis at the time of diagnosis (6). There is considerable variation in the incidence of GC among different geographic regions in the world. Nearly two-thirds of GC occurs in developing countries (7).

The global age-standardized incidence in both men and women is concentrated primarily in Asia (8) and the highest incidence has been reported from some eastern Asian countries such as China, Korea and Japan (9). And regionally, gastric cancer is the first most common cancer in eastern Asia and the highest estimated mortality rates are in this region (8). In Iran, located in Middle East, it is the most frequently diagnosed form of cancer and its trend is increasing (10). Low incidence rates are found in south Asia (11). 

**Figure 1:**
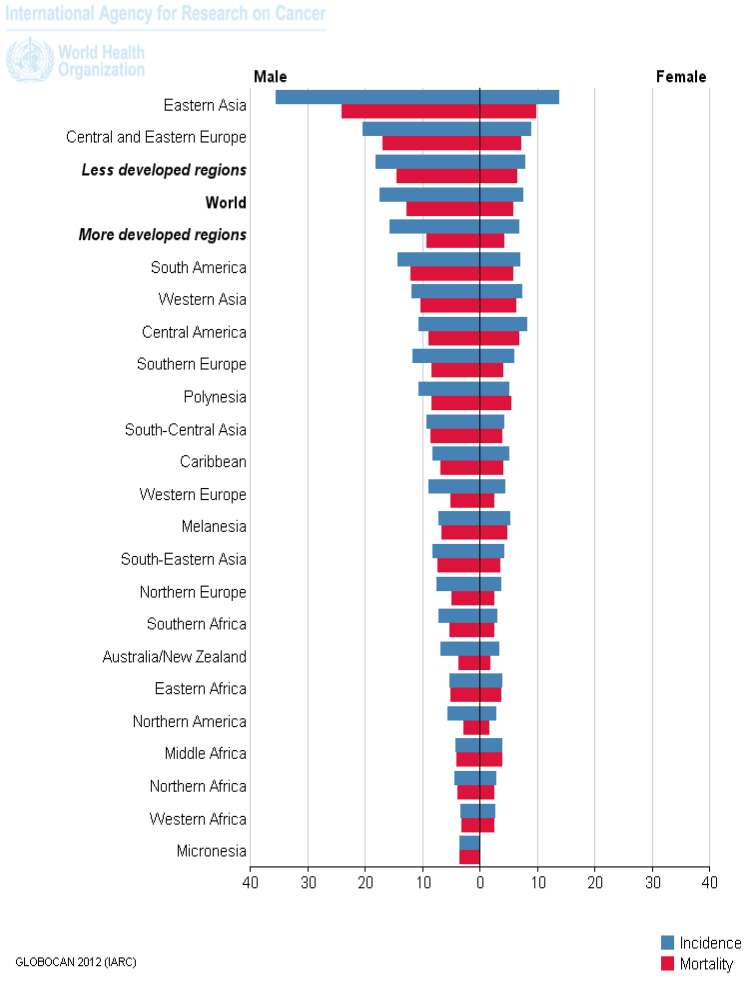
The mortality and incidence of Gastric cancer in the world, according to GLOBOCAN estimation project, 2012.

The age-adjusted rates have been observed to be dramatically falling in all countries including China, Japan. However, the crude rates are estimated to rise substantially between the years 2000 to 2020 (12). Besides, it is predicted that the burden of GC has recently shown decreased incidence and mortality rate (13). The age-standardized rate (ASR) of incidence for Asia in 2012 according to GLOBOCAN estimation was 15.8 and ASR mortality was 11.7 per 100,000 (table 1). After East Asia, the incidence and mortality rate was higher in western Asia, but lower for south-central and southeastern regions of the continent (Figure 1) (14). 

*Helicobacter pylori* (*H pylori*) is on of the GC’s risk factors and countries with high gastric cancer rates typically have a high prevalence of *H. pylori *infection, for instance, in Korea, 90% of asymptomatic adults over the age of 20 years are infected by *H. pylori* (15). On the other hand, the decline in *H. pylori *prevalence is in correspondence to decreasing incidence of GC (16). The incidence of GC is low in some parts of Indonesia (17, 18), which would be due to the infrequency of *H. pylori*. Similar results have been reported in Malaysia, (except for Chinese people in Penang) where the *H. pylori *infection rate is exceptionally low (19). Whether some dietary factor may be playing a role remains unclear.

**2. Colorectal Cancer**

Colorectal cancer is now the third most common malignant disease in Asia (20). In Eastern Asia, countries such as China, Japan, South Korea and Singapore have experienced an increasing incidence in the past decades and among ethnic groups in Asia, the incidence of colorectal cancer is significantly higher among the Chinese (20). A rapid increase in incidence of colorectal cancer has also been reported in Taiwan (21). In Middle East, the incidence of colorectal cancer is increasing (22, 23). 

The mortality of CRC is higher in the less developed regions of the world, reflecting a poorer survival in these regions. Its mortality has been increasing in the last decade in Asian countries, except in Japan and Singapore (20). Other studies reported increasing trend of mortality due to CRC in Korea, China and Iran (24-27). 

**Figure 2:**
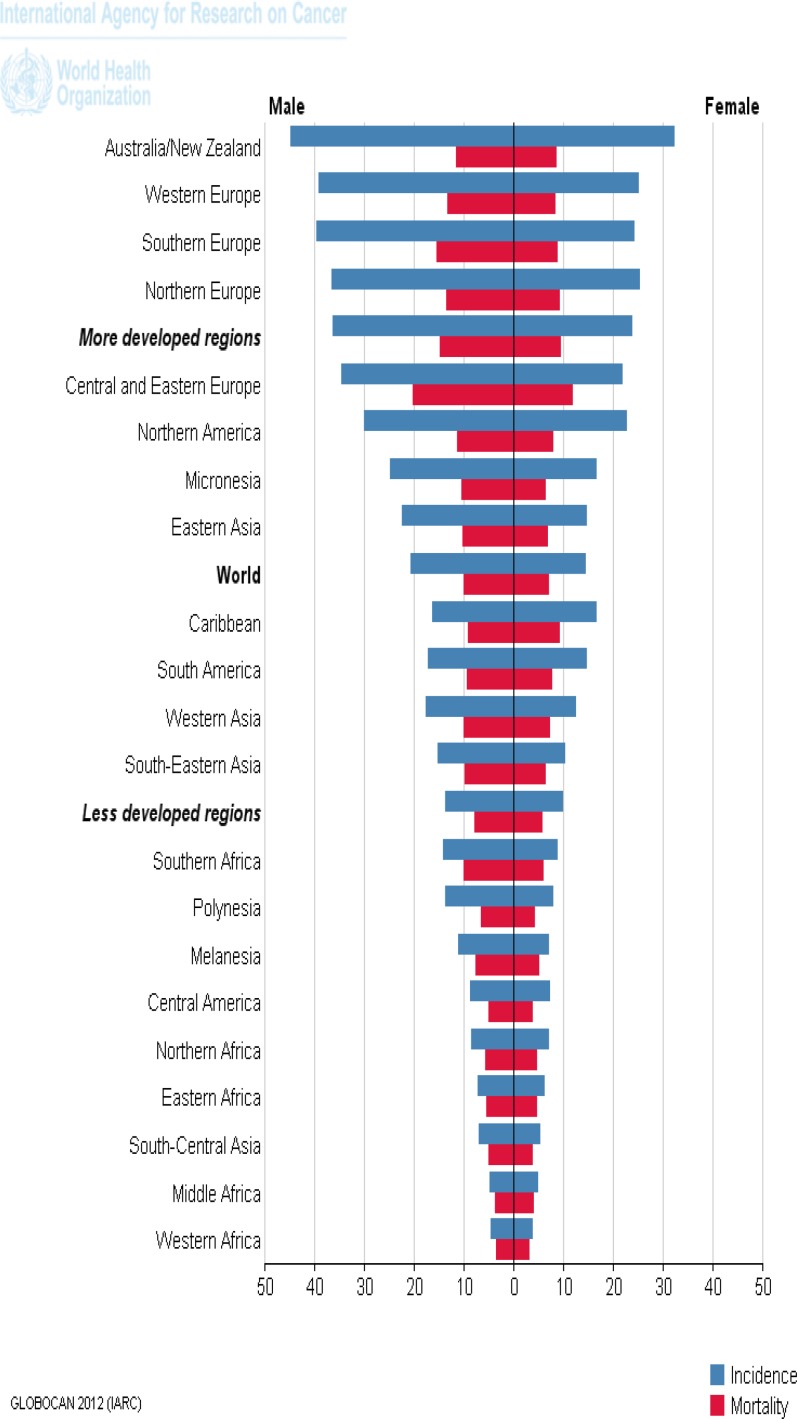
The mortality and incidence of colorectal cancer in the world, according to GLOBOCAN estimation project, 2012

GLOBOCAN estimation project for 2012 indicated that, the ASR incidence for Asia was 13.7 and ASR mortality was 7.2 per 100,000 (table 1). Although the incidence and mortality rate of this cancer are still higher in westernAsia, the ratio of mortality/incidence for Asian regions are higher, which means that the poor survival (figure 2) (14). 

**Table 1:** Estimated Incidence and Mortality for Asian Continent, GLOBOCAN 2012

	Incidence				Mortality			
	Numbers	Crude Rate	ASR	Cumulative Risk	Numbers	Crude Rate	ASR	Cumulative Risk
Gastric Cancer	699954	16.5	15.8	1.77	527074	12.4	11.7	1.22
Colorectal Cancer	607182	14.3	13.7	1.52	331615	7.8	7.2	0.71
Hepatocellular Carcinoma	594431	14.0	13.3	1.46	566886	13.3	12.6	1.35
Esophageal Cancer	340475	8.0	7.7	0.89	298719	7.0	6.7	0.72
Pancreatic Cancer	143363	3.4	3.2	0.34	137251	3.2	3.0	0.32

Crude and ASR rates per 100,000; Cumulative risk; percent; GLOBOCAN 2012, IARC -5.7.2014

According to Asia Pacific cohort studies collaboration, smoking, body mass index and lack of physical activity increased risk of CRC (28). 

Screening can reduce the burden and mortality. In most Asian countries, national health-care systems and health insurance cover only a minority of people (29) and there is little health authority support for colorectal cancer screening and very low public awareness of this emerging epidemic in Asia. 

**3. Hepatocellular Carcinoma:**

**Figure 3:**
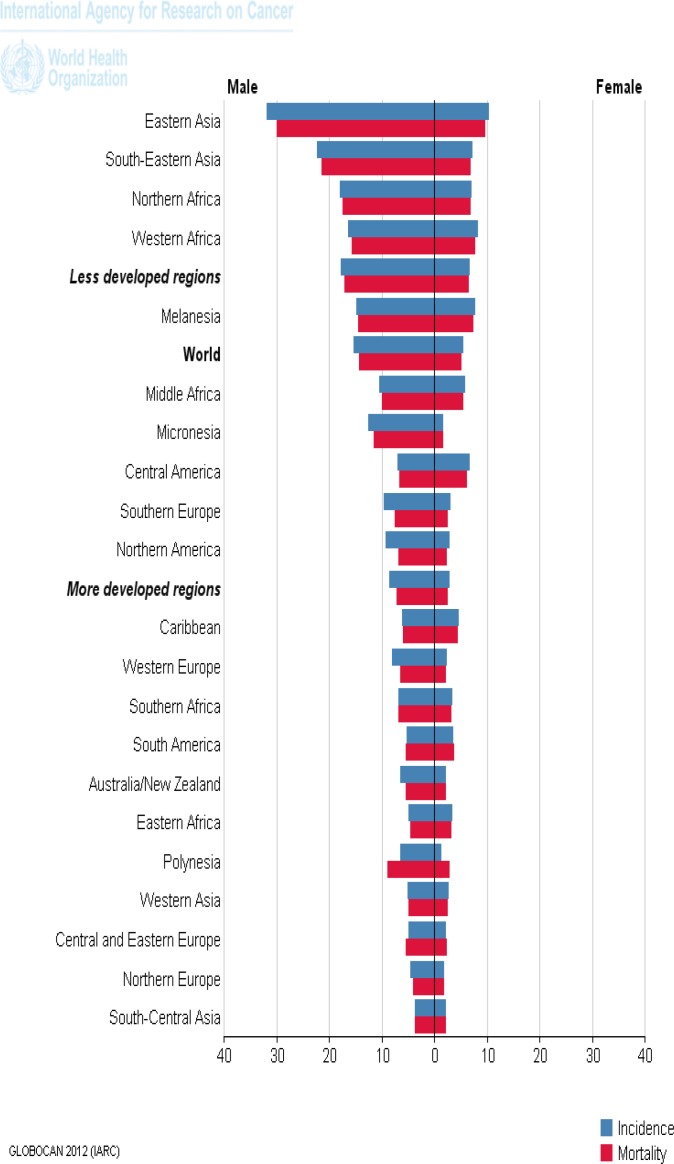
The mortality and incidence of hepatocellular carcinoma in the world, according to GLOBOCAN estimation project, 2012

The distribution of hepatocellular carcinoma (HCC) is heterogeneous with a high prevalence seen in Asia (30) and eighty percent of the burden is borne by countries in Asia and sub-Saharan Africa (31). The regions of high incidence and mortality are eastern and Southeastern Asia (Figure 3) and the highest liver cancer rate in the world is in Qidong, China, based on the cancer registry reporting and another high rate is also reported from Thailand (31). According to GLOBOCAN for 2012, the ASR incidence was 13.3 and ASR mortality was 12.6 per 100,000 (Table 1) (14). 

HCV and HBV are the major etiological agents that lead to the development of HCC (32). The majority of infected people with HBV reside in the HCC high-risk regions of Asia and Africa. In the Asian eastern countries, HBV is the first cause of HCC. In India, HBV is the major risk factors, and Asian countries such as Hong Kong and Taiwan also had high incidence of HBV-related HCC (33). In Japan HBsAg-positive cases of HCC constituted 42% in 1977–1978, but recently reduced (34). In Korea approximately 65–75% of HCC patients are positive for HbsAg (35).

In Iran, the most cause of HCC is HBV and 80% of HCC cases are positive for at least one of the markers of hepatitis B virus (36). Anti-HCV positives are significantly associated with the development of HCC, (25) and the co-infection of hepatitis B and C is associated with a further increased risk of HCC (30).

The other risk factor is aflatoxin (37), which is prevalent particularly in Africa, Southeast Asia and China (38) and most HCC cases due to aflatoxin occur in southeast Asia and China, where populations suffer from both high HBV prevalence and largely uncontrolled exposure to aflatoxin in the food. Alcohol as the other risk factor has important role in low incidence areas than in high incidence areas like Japan (39). The mortality is increasing in China (40, 41) decreasing in Korea and Japan (42, 43) and reaching a plateau in Iran (36). 

**4. Esophageal Cancer:**

**Figure 4:**
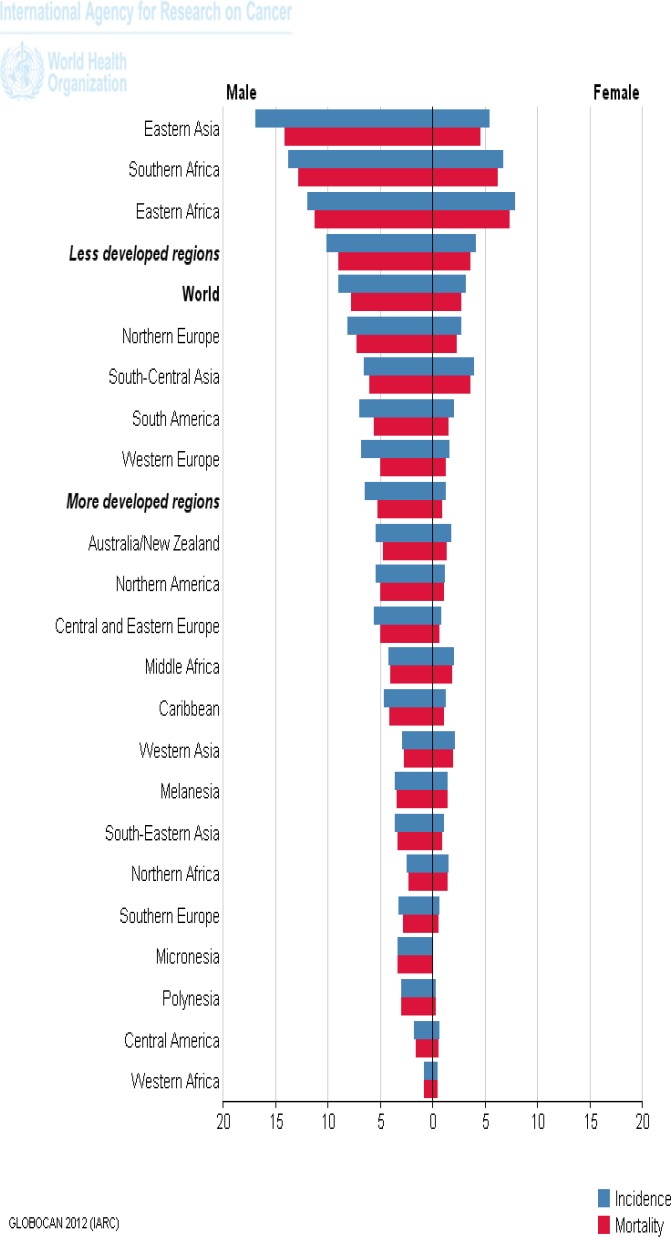
The mortality and incidence of Esophageal cancer in the world, according to GLOBOCAN estimation project, 2012

Esophageal cancer (EC) is one of the most common cancers worldwide (44). Survival rates are very low (45) and its prognosis is poor (46). The incidence and mortality rates show a wide geographical variation with differences between high-and low-risk areas (9, 44). EC is a relatively rare form of cancer and around 80% of the cases worldwide occur in less developed regions but some areas have a higher incidence than others likeChina, Iran, India, Japan, and the region around the Caspian Sea (7). The eastern region and then south-central region have the high incidence and mortality, compared to other parts of continent (figure 4) (14). 

The eastern part of the Caspian littoral area of Iran has the highest incidence of EC in the world (47). In China EC is ranked second in incidence (48). However, recent study revealed that incidence and mortality rates for EC are decreasing due to changes in population, dietary patterns and food preservation methods (49). In Japan a decrease in mortality was observed as well (50). In Iran (which has a high incidence in its Caspian region) the mortality is increasing dramatically (51). According to GLOBOCAN project for Asia in 2012, the ASR incidence was 7.7 and ASR mortality was 6.7 per 100,000 (Table 1) (14).

**5. Pancreatic Cancer:**

Pancreatic cancer is a rapidly fatal cancer with the poorest survival rate of any major malignancy, only with 25-30% five-year survival after surgery and the mortality approaching the incidence (52). 

The mortality rates of pancreatic cancer in developed countries such as Australia and Japan ranged from 6 to 8 per 100,000 in males, and 4 to 6 in females (53). However, in these countries, the mortality rate of pancreatic cancer, have leveled off after an increase (54). 

In some Asian countries, such as Korea and Singapore, the mortality are also high but, not reaching the peak yet (53) and in China the death rate due to pancreatic cancer was rising and the peak mortality might arrive in future (55). In Iran, trend of pancreatic cancer mortality was slightly decreased and is going to be leveled off in recent decade (56, 57). GLOBOCAN estimation for Asia in 2012 showed that ASR incidence was 3.2 and ASR mortality was 3.0 per 100,000 (table 1) (14). 

Pancreatic cancer is one of the diseases that are correlated with industrialization and statistics suggested that majority of deaths occurred in developed countries (1). Smoking, type 2 diabetic mellitus and obesity are widely accepted as the risk factors for pancreatic cancer (58). On the other hand, we face the recent substantial increases in the prevalence of cigarette smoking; type 2 diabetic mellitus, and obesity in Asian countries and recent studies in Asia revealed that smoking, obesity, and diabetes are important and potentially risk factors for pancreatic cancer in populations of the Asia-Pacific region (59, 60).

## Conclusion

Asia’s burden of GI cancer is predicted to increase. Liver, gastric and esophageal cancers are relatively common in Asia. Three fourths of worldwide liver cancer cases in males and two thirds in females occur in the fifteen Asian countries and China alone has more than half of newly diagnosed liver cancer cases in the world. The prevalence of liver cancer is still high in Asia (30) and the main challenge is the high prevalence of chronic hepatitis (61).Although, HBV vaccination in these areas like China should be the major preventive tactic (31). Gastric cancer is the other GI cancer, rising in Asia. Although age-adjusted rate of GC is falling, the absolute number of cases and deaths are rising because of the increasing size and age of the world population, especially in the developing countries. Japanese experience revealed that, the availability of screening for early detection in high-risk areas has led to a decrease in mortality of this fatal cancer (62). The essential strategy for prevention and control the burden of GC in Asian countries with highest incidence would be focus on controlling *H. pylori* infection and improving educational levels, advocating healthy diet and also cost-effective early detection programs (63, 64). 

Colorectal cancer is increasing in Asian population; the changing epidemiology is very worrying as the rising incidence in Asia (65). The increasing rate means that we need to take action immediately to prevent colorectal cancer and to diagnose the disease at the early stages by the cost-effectiveness of screening program (66). Esophageal cancer also occurs disproportionately in Asia, greater than 70% of new cases in males and females occur in the fifteen Asian countries. Low general awareness about the symptom of EC and delay in diagnosis of EC due to lack of a national comprehensive system for early detection of this cancer lead to diagnosis of EC in older ages and the subsequent higher mortality rates. Therefore, conducting a program to increase general awareness of known and probable risk factors of EC may be helpful to reduce EC incidence, especially in high incidence area. 

Finally, the last but not the least GI cancer in Asia, is pancreatic cancer. However, the rate is not too high.Recent studies revealed that smoking, obesity, and diabetes are important and potentially risk factors, as similar as the western countries, for pancreatic cancer in populations of the Asia-Pacific region (76, 77). Thus, the activities to prevent them can be lead to reduction in the incidence and mortality of this cancer in Asian countries. 

Whereas, the limitation of the incidence and mortality data due to incomplete registration sources for low income countries, the burden of GI cancer is increasing in Asia because of aging, growth of the population and the risk factors including smoking, obesity, changing lifestyle and high prevalence of *H.pylori,* HBV and HCV. On the other hand, there are many people in the developing countries inside Asia who have no health insurance and many of them are too poor to go for screening tests, early detection or medical treatments. Thus, it is important for the health organizations and governments in each country to recognize these groups and reduce the incidence and mortality of GI cancers, using simple and economic screening test, vaccination and changing risk factors such as smoking, diet and lifestyle by education programs.
